# Prognostic role of C-reactive protein-albumin-lymphocyte (CALLY) index in gastrointestinal malignancies: a systematic review and meta-analysis

**DOI:** 10.1186/s12876-026-04793-7

**Published:** 2026-04-10

**Authors:** Yaser Rayyan, Nada Odeh, Laith Theeb, Yazeed Y. Alajlouni, Mohammad Alzoubi, Noor F. Al-Assaf, Layan A. Jaber, Hashim M. AlHammouri, Abdullah H. Alzghoul, Abdallah M. Khamaiseh, Ayham S. Hamdan, Ahmad M. Alhaj

**Affiliations:** 1 School of Medicine, The University of Jordan, Amman, Jordan; 2 Department of Internal Medicine, Section of Gastroenterology, Jordan University Hospital, Queen Rania Al-Abdullah St., Amman, 11942 Jordan

**Keywords:** CALLY index, Gastrointestinal malignancies, Meta-analysis, Postoperative complications, Prognostic biomarker, Survival analysis

## Abstract

**Introduction:**

Gastrointestinal (GI) malignancies are a major cause of cancer-related morbidity and mortality. While prognostic tools such as TNM staging and serum biomarkers are widely used, they have limitations. The C-reactive protein-albumin-lymphocyte (CALLY) index, an integrated marker of inflammation, nutrition, and immunity, has emerged as a potential prognostic tool. However, its prognostic value across GI malignancies remains unclear. This systematic review aims to assess the prognostic significance of the CALLY index in GI malignancies, with a primary focus on survival outcomes and a secondary assessment of its association with postoperative complications.

**Methodology:**

Relevant literature was screened through December 2024 to identify studies assessing the prognostic significance of the CALLY index in GI malignancies. Outcomes included overall survival (OS), cancer-specific survival (CSS), disease-free survival (DFS), recurrence-free survival (RFS), recurrence rates, and postoperative complications. Eligible studies underwent qualitative synthesis, while meta-analysis was conducted for gastric cancer only. Relative risks (RRs) were pooled using a fixed- or random-effects model based on heterogeneity (I² > 50%). Subgroup analyses were performed based on CALLY cutoff values and TNM stage. Publication bias was assessed using funnel plots and Egger’s test.

**Results:**

A total of 21 studies (8,600 patients) met inclusion criteria. Across all GI malignancies, higher CALLY values, regardless of cutoff variations (range: 0.369–6.96), were consistently associated with improved OS, CSS, DFS, and RFS, while lower values correlated with increased recurrence rates and higher postoperative complication rates. In gastric cancer, a higher CALLY index was associated with improved 5-year OS (RR = 0.81, *p* < 0.00001) and lower major postoperative complications (RR = 1.48, *p* = 0.02). Subgroup analysis suggested that a CALLY cutoff < 3 and TNM stage IV inclusion contributed to heterogeneity. No significant publication bias was detected.

**Conclusion:**

The CALLY index appears to be a promising independent prognostic biomarker in GI malignancies, with higher values associated with improved survival and fewer postoperative complications. In gastric cancer specifically, meta-analysis confirms its predictive value for 5-year overall survival and major postoperative outcomes. Larger prospective studies are warranted to establish standardized cutoff points and to optimize its clinical utility across diverse patient populations.

**Supplementary Information:**

The online version contains supplementary material available at 10.1186/s12876-026-04793-7.

## Introduction

Gastrointestinal (GI) malignancies, encompassing cancers of the esophagus, stomach, liver, gallbladder, pancreas, and colorectal, are among the most common cancers worldwide, representing a significant global health burden. According to recent global cancer statistics, GI cancers account for nearly a quarter (24.6%) of all new cancers globally, contributing to more than a third (34.2%) of all cancer-related mortalities [1]. Despite advancements in diagnostic techniques and treatment modalities, the survival outcomes for patients with gastrointestinal malignancies remain unsatisfactory [2–5]. This underscores the need for developing more effective therapeutic strategies, driven by novel approaches in diagnosis, prognosis, and treatment methods. To address this, a variety of prognostic markers have been developed and are currently being utilized in clinical practice. Among these, the Tumor-Node-Metastasis (TNM) staging system remains the most widely employed prognostic tool for GI malignancies [6]. Serum-based biomarkers such as carcinoembryonic antigen (CEA) and carbohydrate antigen 19 − 9 (CA 19 − 9), as well as molecular markers like microsatellite instability (MSI), KRAS, NRAS, and BRAF mutations, further enhance prognostication by providing insights into tumor behavior and response to therapy [7, 8]. Finally, inflammatory markers, such as the neutrophil-to-lymphocyte ratio (NLR), platelet-to-lymphocyte ratio (PLR), systemic inflammatory response index (SIRI), along with nutritional indices such as Prognostic Nutritional Index and Glasgow Prognostic Score, are increasingly being used to predict survival, reflecting the critical link between systemic inflammation, nutrition, and tumor progression [9].

However, these prognostic markers have notable limitations, as they typically assess individual aspects, such as inflammation level, nutritional status, or immune function, without accounting for the complex interrelationships among these factors in cancer progression and prognosis. This limitation highlights the need for more integrative markers that encompass these dimensions.

One such marker is the C-reactive protein-albumin-lymphocyte (CALLY) index, a novel prognostic biomarker developed by Iida et al. [10]. The CALLY index integrates markers of nutritional, inflammatory, and immunological statuses, all of which are key determinants of cancer prognosis as supported by existing literature [11, 12].

This novel biomarker has been shown to be an independent prognostic factor for survival outcomes in GI cancers [6, 13–15]. However, these studies have primarily focused on specific cancer subtypes, limiting its ability to generalize findings across the broader spectrum of GI cancers. Furthermore, the lack of systematic analyses limits the clear understanding of the prognostic value of the CALLY index and its application in clinical practice. Despite a growing recognition of the association between systemic inflammation, nutrition, and immune status in cancer prognosis, there remains a gap in assessing whether the CALLY index can serve as a standardized and reliable prognostic tool.

This systematic review aims to provide a comprehensive evaluation of the prognostic value of the CALLY index across GI malignancies. It synthesizes existing research on the association between the CALLY index and survival outcomes, assessing its potential as a standardized prognostic tool. While postoperative complications were examined when reported, they were considered separately from prognostic outcomes to provide additional clinical context.

## Materials and methods

### Literature search strategy

A comprehensive search was conducted across PubMed, Scopus, and Web of Science from inception to December 31, 2024. The search strategy was tailored to each database using a combination of keywords, MeSH terms, and Boolean operators. The citation lists of all identified studies were manually screened for additional relevant articles. Detailed search syntax for all databases is available in Supplementary Material (S1 File).

### Inclusion criteria for study selection

Studies included in this review were original cohort studies, either prospective or retrospective, or secondary analyses of prospective cohorts, published as full-text articles in peer-reviewed journals. Eligible studies focused on adult patients who were diagnosed with gastrointestinal malignancies, including colorectal, gastric, esophageal, pancreatic, and hepatobiliary cancers. The primary focus was on studies evaluating the CALLY index, a composite score derived from CRP, albumin, and lymphocyte count. Variations in its calculation or threshold were accepted if justified by the study. Included studies had to report at least one prognostic outcome, such as overall survival (OS), cancer-specific survival (CSS), disease-free survival (DFS), recurrence-free survival (RFS), or recurrence rates, including early recurrence within six months; analyzed using standard statistical methods, including Kaplan-Meier analysis or Cox proportional hazards models.

### Follow-up and outcome measures

Studies were required to have a minimum follow-up duration of six months for primary outcomes or provide a median follow-up duration. Studies that focused on early recurrence (less than six months) or secondary outcomes such as postoperative complications were also included, regardless of the specified follow-up duration. Incomplete follow-up data were accepted if outcomes were reported using validated statistical methods and the implied follow-up was clinically plausible, aligning with the expected progression of malignancy. The CALLY index had to be measured either before the primary disease-altering intervention, such as surgery or transarterial chemoembolization (TACE), or after preparatory or bridging interventions, including neoadjuvant chemotherapy, radiotherapy, or procedural interventions such as stent placement, but prior to the primary therapeutic procedure. The primary comparisons of interest were between high and low CALLY index groups.

### Exclusion criteria

Excluded studies included case-controls, cross-sectionals, reviews, and case reports. Conference abstracts, editorials, or preprints were excluded due to insufficient methodological detail, incomplete outcome reporting, and lack of peer review, which may compromise data reliability and reproducibility. Lastly, studies focusing on mixed cancer types without separate results for gastrointestinal malignancies were deemed ineligible.

### Study selection process

**Fig. 1:**
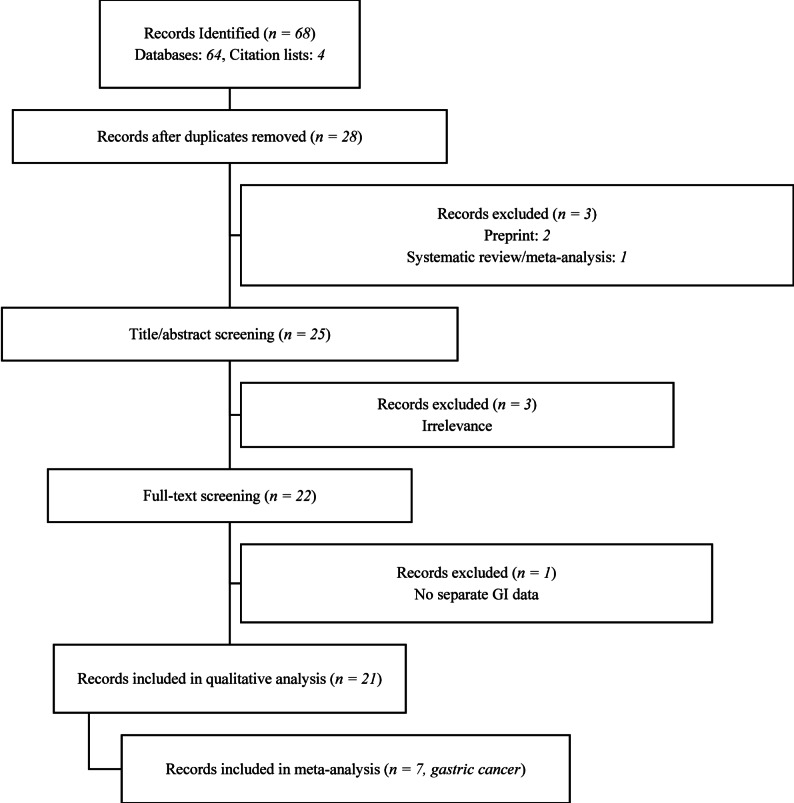
PRISMA flow diagram of study selection

The study selection process is summarized in Fig. 1. Two independent reviewers (N.O, M.A) assessed the titles and abstracts of all identified studies against eligibility criteria. Full-text articles of relevant studies were then retrieved and examined by the same reviewers for final inclusion. Discrepancies were resolved through discussion.

### Quality assessment

Methodological quality was assessed using the Newcastle-Ottawa Scale (NOS), a nine-point scale evaluating selection, comparability, and outcome assessment [16]. Studies scoring more than six points were classified as high quality. Assessments were conducted independently by two investigators (N.F.A, H.M.A), with a third-party reviewer (N.O) consulted to resolve any disagreements. Notably, all included studies scored seven or higher. Detailed NOS scores are provided in Supplementary Material (S2 File).

### Data extraction

Each study was independently extracted by two investigators (L.A.J, Y.Y.A, A.H.A, A.M.K, A.S.H, A.M.A), collecting key study characteristics, including first author’s last name, year of publication, country of origin, study design (prospective or retrospective), setting (single-center or multicenter), and population characteristics such as cancer type, TNM stage, age, and sex distribution. Data related to the CALLY index included its calculation method, timing of measurement, and cutoff points. Outcome measures focused on survival and recurrence, with number of events in the low CALLY and high CALLY groups, and effect estimates such as hazard ratios, odds ratios, confidence intervals, and p-values extracted. Postoperative complications and their associations with the CALLY index were also recorded when applicable. Major postoperative complications were defined as grade III or above on Clavien-Dindo system. Inter-reviewer agreement was assessed using Cohen’s Kappa coefficient (k = 0.93). A Kappa value > 0.80 was considered indicative of excellent agreement. Discrepancies were resolved by consulting a third reviewer (N.O).

### Statistical analysis

A meta-analysis was conducted for the seven studies on gastric cancer because it was the only malignancy with a sufficient number of studies reporting comparable survival outcomes and hazard ratios, assessing the prognostic significance of the CALLY index. Hazard ratios (HRs) were extracted and treated as relative risks (RRs) for consistency in effect size interpretation. A fixed-effects model (FEM) was applied when heterogeneity was low, while a random-effects model (REM) was used when substantial heterogeneity was present (I2 > 50%). Pooled HRs (treated as RRs) with 95% confidence intervals (CIs) were calculated, and forest plots were generated for visualization. All statistical analyses, including publication bias funnel plots, were performed using Review Manager (RevMan) version 5.4., with plot symmetry and Egger’s test p-value < 0.05 indicating a low risk of bias. For other gastrointestinal malignancies, the limited number of eligible studies, heterogeneity in outcome definitions and included populations, and inconsistent reporting formats precluded quantitative synthesis. Therefore, findings for non-gastric malignancies were summarized descriptively.

### Ethics approval and consent to participate

This study is a systematic review and meta-analysis of previously published studies. No new human participants were involved, and no individual-level identifiable data were accessed. Therefore, ethical approval and informed consent were not required.

## Results

### Overview of included studies

A total of 21 studies met the inclusion criteria, encompassing 8,600 patients with various gastrointestinal malignancies. These studies were published between 2021 and 2024, with the majority conducted in Japan (*n = 17*). The prognostic significance of the CALLY index was evaluated in gastric (*n* = 7), colorectal (*n* = 4), esophageal (*n = 3*), hepatocellular (*n = 3*), pancreatic (*n = 2*), and biliary tract cancers (*n = 2*). Sample sizes ranged from 99 to 1,260, with a male predominance observed in most studies (median: 69.5%). A summary of study characteristics is presented in Table 1.

**Table 1:** Summary of included studies

First author, Year	Country	Study Period	Study Design	Cohort type	Cancer Type	TNM Stage	Study Size	Age (Mean/ Median, years)	Male %
Aoyama, Hashimoto et al., 2024 [17]	Japan	2005–2020	Retrospective cohort, single-center	Single cohort	Esophageal	IB – III	180	69 (Median)	86%
Aoyama, Maezawa et al., 2024 [18]	Japan	2005–2020	Retrospective cohort, single-center	Single cohort	Gastric	I-III	259	70 (Median)	71%
Feng et al., 2024 [15]	China	2013.1–2015.12	Retrospective cohort, single-center	Single cohort	Esophageal	I-III	318	59.4 (Mean)	67%
Fukushima et al., 2024 [19]	Japan	2010.5–2017.12	Retrospective cohort, Multicenter	Single cohort	Gastric	I-III	826	68 (Median)	72%
Furukawa et al., 2023 [20]	Japan	2000.2–2018.4	Retrospective cohort, single-center	Single cohort	Colorectal	IV	183	65 (Mean)	69%
Hashimoto et al., 2024 [21]	Japan	2013.12–2017.11	Prospective cohort, single-center	Single cohort	Gastric	I-III	459	NOS	65%
Iida et al., 2022 [10]	Japan	2011.1–2013.12	Retrospective cohort, Multicenter	DC	HCC	I-IV	384	69.6 (Mean)	77%
				VC			267	68.3 (Mean)	76%
Kawahara et al., 2024 [14]	Japan	2013.1–2022.12	Retrospective cohort, single-center	Single cohort	Pancreatic	I-III	461	71 (Median)	53%
Kosaka et al., 2022 [22]	Japan	2009.1–2020.12	Retrospective cohort, Multicenter	Single cohort	ICC	I-IV	227	72 (Median)	70%
Ma et al., 2024 [23]	Japan	2008–2018	Retrospective cohort, single-center	Single cohort	Esophageal	I-IV	146	69 (Median)	84%
Matsui et al., 2024 [24]	Japan	2012.1–2019.12	Retrospective cohort, single-center	Single cohort	Pancreatic	I-III	307	NOS	54%
Müller et al., 2021 [25]	Germany	2010.1–2020.11	Retrospective cohort, single-center	Single cohort	HCC	N/A	280	69.5 (Median)	84%
Nakashima et al., 2024 [13]	Japan	2011.1–2019.10	Retrospective cohort, single-center	Single cohort	Gastric	I-III	175	70 (Median)	68%
Okugawa et al., 2024 [26]	Japan	2000–2011	Retrospective cohort, single-center	Single cohort	Gastric	I-IV	426	67.5 (Median)	71%
Sakurai et al., 2024 [27]	Japan	2014.1–2020.12	Retrospective cohort, single-center	Single cohort	Gastric	I-IV	563	NOS	61%
Shiraishi et al., 2024 [28]	Japan	2016.4–2021.12	Retrospective cohort, Multicenter	Single cohort	Colorectal	I-IV	263	NOS	57%
Takeda et al., 2024 [29]	Japan	2010.2–2017.12	Retrospective cohort, single-center	Single cohort	Colorectal	II, III	578	69 (Median)	60%
Tsunematsu et al., 2022 [30]	Japan	2002.4–2019.6	Retrospective cohort, single-center	DC	CC	I-IV	143	68 (Median)	46%
			Retrospective cohort, Multicenter	VC			99	NOS	NOS
Yang et al., 2023 [6]	China	2012.1–2020.10	Secondary analysis of a prospective cohort, Multicenter	Single cohort	Colorectal	I-IV	1260	60 (Median)	61%
Yasuda et al., 2024 [31]	Japan	2000.1–2022.12	Retrospective cohort, single-center	Single cohort	HCC	I-IV	112	NOS	94%
Zhang et al., 2023 [32]	China	2013.5–2018.12	Retrospective cohort, Multicenter	DC	Gastric	I-IV	684	59 (Mean)	70%
				VC			290	61 (Mean)	72%

*HCC* Hepatocellular Carcinoma, *ICC* Intrahepatic Cholangiocarcinoma, *CC* Cholangiocarcinoma, *D* Discovery Cohort, *VC* Validation Cohort, *NOS* Not Otherwise Specified, *N/A* Not Applicable

### CALLY index calculation and cutoff values

All included studies calculated the CALLY index using the equation: Albumin (g/dl) × Lymphocyte (/µl) / (CRP (mg/dl) × 10^4^). However, cutoff values varied, ranging from 0.369 to 6.96, with the most commonly used cutoff of 5 reported in three studies (Table 2). Cutoff determination methods included receiver operating characteristic (ROC) curve analysis (*n = 17*), previously established thresholds based on prior literature or survival rates at 3 and 5 years (*n = 2*), optimal stratification using the *‘survminer’* and *‘survival’* R packages (*n = 1*), and restricted cubic spline (RCS) modeling (*n = 1*).

**Table 2:** Prognostic outcomes of the CALLY index in different gastrointestinal cancers

Esophageal	CALLY cutoff value	Outcome Assessed	HR/OR	95% CI	*p*-value	Favorable Prognostic Group
Aoyama, Hashimoto et al., 2024 [17]	5	OS	HR 2.31	1.416–3.767	< 0.001	CALLY-high
		RFS	HR 2.093	1.384–3.165	< 0.001	
Feng et al., 2024 [15]	1.7	CSS	HR 0.368	0.268–0.506	< 0.001	CALLY-high
Ma et al., 2024 [23]	2.4	OS	HR 3.86	2.03–7.34	< 0.0001	CALLY-high
	4.56	DFS	HR 2.35	1.28–4.31	0.006	
Colorectal						
Furukawa et al., 2023 [20]	4	OS	HR 0.6	0.39–0.94	0.03	CALLY-high
		DFS	HR 0.72	0.49–1.05	0.09	
Shiraishi et al., 2024 [28]	0.369	OS	NOS	NOS	NOS	CALLY-high
		RFS				
Takeda et al., 2024 [29]	2	OS	HR 2.79	1.32–5.92	0.007	CALLY-high
		DFS	HR 2.16	1.25–3.75	0.006	
Yang et al., 2023 [6]	1.47	OS	HR 0.45	0.36–0.56	< 0.001	CALLY-high
HCC						
Iida et al., 2022 [10]						
DC	5	OS	HR 1.7	1.21–2.38	0.002	CALLY-high
		RFS	HR 1.41	1.08–1.83	0.011	
VC		OS	HR 1.81	1.21–2.71	0.003	
		RFS	HR 1.56	1.12–2.15	0.007	
Müller et al., 2021 [25]	1	OS	HR 1.5	1.1–2.1	0.008	CALLY-high
Yasuda et al., 2024 [31]	2.8	VER	OR 4.3	1.41–13.1	0.007	CALLY-high
Pancreatic						
Kawahara et al., 2024 [14]	1.9	OS	HR 1.772	1.362–2.305	< 0.001	CALLY-high
		RFS	HR 1.289	1.006–1.652	0.045	
Matsui et al., 2024 [24]	3	OS	HR 1.56	1.1–2.22	0.012	CALLY-high
		DFS	HR 1.43	1.01–2.01	0.041	
BTCs						
Kosaka et al., 2022 [22]	3	DFS	HR 2.937	1.991–4.334	< 0.001	CALLY-high
		CSS	HR 3.038	1.951–4.73	< 0.001	
Tsunematsu et al., 2022 [30]						
DC	3.5	OS	HR 2.07	1.11–3.89	0.02	CALLY-high
		DFS	HR 2.13	1.15–3.86	0.02	
VC		OS	NOS	NOS	NOS	
		DFS				
Gastric						
Aoyama, Maezawa et al., 2024 [18]	5	OS	HR 1.791	1.067–3.009	0.028	CALLY-high
		RFS	HR 1.1776	1.102–2.865	0.018	
Fukushima et al., 2024 [19]	2	OS	HR 2.02	1.18–3.46	0.01	CALLY-high
		RFS	HR 1.88	1.11–3.17	0.02	
Hashimoto et al., 2024 [21]	3.28	OS	HR 1.96	1.19–3.23	0.008	CALLY-high
		RFS	HR 1.69	1.06–2.7	0.03	
Nakashima et al., 2024 [13]	6.96	OS	HR 3	1.31–6.93	0.01	CALLY-high
		DFS	HR 2.18	1–4.76	0.05	
Okugawa et al., 2024 [26]	4.93	OS	HR 2.57	1.62–4.07	0.0001	CALLY-high
		DFS	HR 1.76	1.01–3.05	0.045	
Sakurai et al., 2024 [27]	1.19	OS	HR 1.82	1.24–2.69	0.002	CALLY-high
		CSS	HR 1.93	1.19–3.23	0.007	
Zhang et al., 2023 [32]						
DC	1.12	OS	HR 0.7	0.55–0.9	0.004	CALLY-high
VC			HR 0.61	0.42–0.9	0.011	

*OS* Overall Survival, *CSS* Cancer-specific Survival, *DFS* Disease-free Survival, *RFS* Relapse/Recurrence-free Survival, *VER* Very Early Recurrence, *HR* Hazard Ratio, *OR* Odds Ratio, *NOS* Not Otherwise Specified

### Prognostic role of the CALLY index by cancer type

Across the included studies, a higher CALLY index was generally associated with favorable outcomes. Multivariate analyses in several studies confirmed the independent prognostic value of CALLY for OS, CSS, DFS, RFS, and recurrence rates. A detailed breakdown is provided in Table 2.

### Esophageal cancer

All three studies on esophageal cancer (EC) assessed patients undergoing surgical resection. Ma et al. found that a lower CALLY index was associated with significantly worse post-resection DFS (5-year DFS: 10.7% vs. 18.5%, *p* = 0.0009) and OS (5-year OS: 11.4% vs. 22.5%, *p* = 0.0001). Additionally, postoperative surgical site infection (SSI) was significantly more frequent in patients with a low CALLY index (*p* = 0.005). Multivariate analysis confirmed a low CALLY index as an independent predictor of postoperative SSI (HR 11.6, 95% CI 2.32–212, *p* < 0.0001) [23].

Aoyama and Hashimoto et al. found that a lower CALLY index was associated with significantly worse post-resection OS (*p* < 0.001) with 3-year OS at 50% vs. 75.9% and 5-year OS at 42.6% vs. 66.6%. Furthermore, Aoyama and Hashimoto et al. found that a lower CALLY index was associated with significantly lower post-resection RFS (*p* < 0.001) with 3-year RFS at 31.1% vs. 57.8% and 5-year RFS at 27.3% vs. 50.8%. The pattern of recurrence was also analyzed, with the low CALLY index group experiencing significantly higher rates of Lymphatic and hematological recurrence (*p* = 0.012, < 0.001; respectively). Unlike Ma et al., the incidence of postoperative SSI did not significantly vary by the CALLY index; however, the incidence of anastomotic leakage was significantly higher in the low CALLY group (40.25 vs. 27.5%, *p* = 0.030) [17].

Lastly, Feng et al. focused exclusively on patients with squamous EC who underwent radical resection. They found that patients with a low CALLY index experienced significantly worse 5-year post-resection CSS (21.8% vs. 62.6%, *p* < 0.001) [15].

### Colorectal cancer

Four studies evaluated the prognostic significance of the CALLY index in colorectal cancer (CRC) across different patient populations. Furukawa et al. assessed patients with colorectal liver metastases undergoing hepatectomy and found that a lower CALLY index was associated with significantly worse post-resection DFS (*p* = 0.01) and OS (*p* < 0.01). Specifically, 3-year DFS was 21.4% in the low CALLY group vs. 39.4% in the high CALLY group. OS rates were also lower in the low CALLY group, with 3-year OS at 62.9% vs. 76.1% and 5-year OS at 42.3% vs. 64.9%. Postoperative complications were more frequent in the low CALLY group (29% vs. 11%, *p* < 0.01) [20].

Shiraishi et al. examined CRC patients who underwent resection following colonic stenting for obstructive disease [28]. A lower CALLY index was not significantly associated with post-resection OS (*p* = 0.092) or RFS (*p* = 0.971) but was linked to a higher incidence of postoperative complications (30.6% vs. 18.5%, *p* = 0.039). Multivariate analysis confirmed a low CALLY index as an independent predictor of postoperative complications (OR 1.961, 95% CI 1.013–3.795, *p* = 0.045).

Takeda et al. analyzed patients undergoing curative resection for CRC and found that a lower CALLY index was associated with significantly worse post-resection DFS (5-year DFS: 60.7% vs. 77.8%, *p* < 0.001) and OS (5-year OS: 75.9% vs. 91.6%, *p* < 0.001) [29]. Similarly, Yang et al. reported that a higher CALLY index was significantly associated with a lower mortality risk among CRC patients [6] (Table 2).

### Hepatocellular carcinoma

Despite differences in therapeutic strategies, three studies assessed the prognostic significance of the CALLY index in patients with hepatocellular carcinoma (HCC). Yasuda et al. focused on HCC patients with tumor burdens exceeding the Milan criteria who underwent curative-intent resection. They found that a low CALLY index was an independent predictor of very early recurrence (VER), defined as recurrence within six months post-resection [31] (Table 2).

Iida et al. examined HCC patients undergoing hepatectomy, regardless of primary tumor characteristics, in both discovery and validation cohorts [10]. In both cohorts, a low CALLY index was associated with significantly worse post-resection 5-year OS (discovery: 46% vs. 71%, validation: 48% vs. 73%; *p* < 0.001) and 5-year RFS (discovery: 24% vs. 38%, validation: 26% vs. 43%; *p* < 0.001).

Müller et al. evaluated HCC patients treated with TACE and found that a lower CALLY index was associated with significantly worse post-TACE OS (median: 9 vs. 24 months, *p* < 0.0001; 3-year OS: 7.5% vs. 18.5%) [25].

### Pancreatic cancer

Two studies evaluated the prognostic significance of the CALLY index in pancreatic cancer patients undergoing surgical resection. Kawahara et al. reported that a lower CALLY index was associated with significantly worse post-resection OS (median: 22.1 vs. 37.9 months, *p* < 0.001) and RFS (median: 12.4 vs. 16.2 months, *p* = 0.001). The 5-year OS and RFS rates were also lower in the low CALLY group (OS: 46% vs. 71%, *p* < 0.001; RFS: 24% vs. 38%, *p* < 0.001). However, postoperative complications did not differ significantly between the two groups (*p* = 0.555) [14]. Similarly, Matsui et al. found that a lower CALLY index was associated with significantly worse DFS (median: 13.8 vs. 32.0 months, *p* < 0.001; 5-year DFS: 20.1% vs. 37.7%) and OS (median: 23.9 vs. 61.3 months, *p* < 0.001; 5-year OS: 28.3% vs. 50.7%) following curative resection [24].

### Cholangiocarcinoma

In patients with cholangiocarcinoma undergoing surgical resection, a lower CALLY index was associated with significantly worse survival outcomes. Kosaka et al. studied patients with intrahepatic cholangiocarcinoma following hepatectomy and found that those with a low CALLY index had markedly poorer post-resection CSS (median: 27.2 vs. 77.5 months, *p* < 0.001) and DFS (median: 8.6 vs. 42.9 months, *p* < 0.001) [22].

Tsunematsu et al. evaluated patients with distal cholangiocarcinoma undergoing pancreaticoduodenectomy in both discovery and validation cohorts [30]. In both cohorts, low CALLY index was associated with significantly worse post-resection DFS (discovery cohort: 19.5% vs. 48.2%, *p* < 0.01; validation cohort: 13.1% vs. 27.8%, *p* = 0.03) and OS (discovery: 23% vs. 58.9%, validation: 14.8% vs. 36.1%; *p* < 0.01). Although discovery cohort revealed that postoperative complications were more frequent in the low CALLY group (41% vs. 31%), the difference was not statistically significant (*p* = 0.24).

### Meta-analysis of the prognostic role of CALLY index in gastric cancer

#### Comparison of the 5-year overall survival

**Fig. 2:**
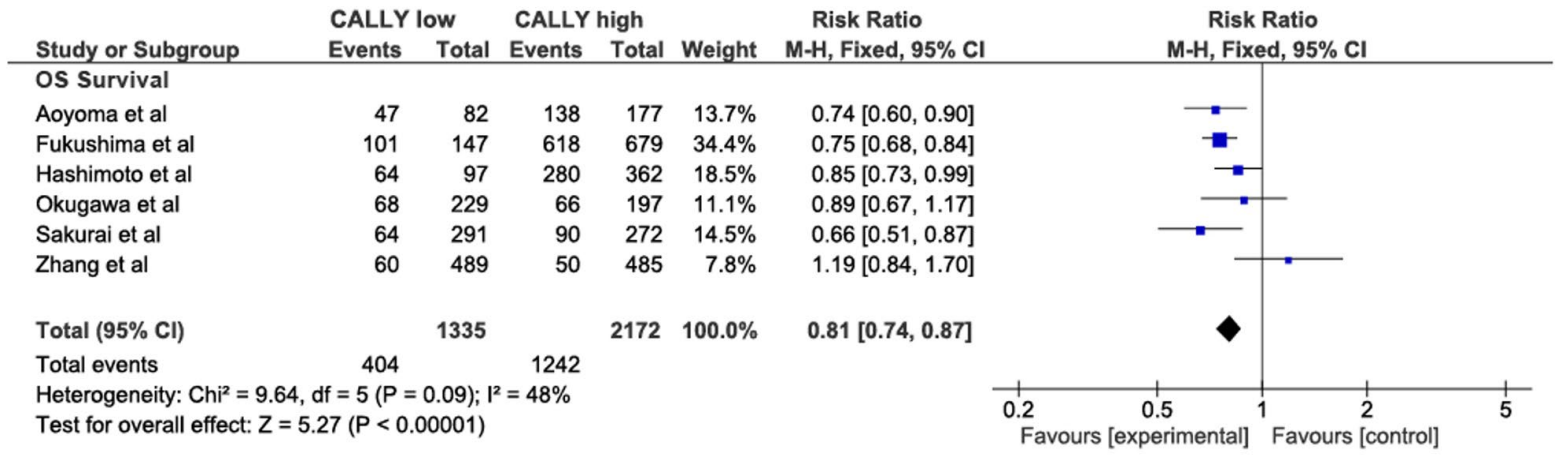
Comparison of the patients’ 5-year overall survival (OS)

There were 6 studies [18, 19, 21, 26, 27, 32] included in the assessment of OS regarding the CALLY index. The forest plot revealed that the lower CALLY index group had lower survival than the high CALLY index group (RR = 0.81, 95% CI 0.74–0.87, *P* < 0.00001; Fig. 2). Due to the moderate heterogeneity (I2 = 48%) in the studies included in the meta-analysis, a subgroup analysis was performed according to TNM stages, CALLY cutoff values, and surgical technique. It was found that subgroup heterogeneity was high in the “I-IV TNM stage” group (RR = 0.87, 95% CI 0.64–1.20, *P* = 0.41, I2 = 70%), and the “CALLY index cutoff < 3” group (RR = 0.82, 95% CI 0.63–1.06, *P* = 0.13, I2 = 73%), and neither result was significant. A subgroup analysis of cohorts in which all patients underwent gastrectomy, including all the studies except Zhang et al., revealed significant results with no heterogeneity (RR = 0.78, 95% CI 0.72–0.84, *P* < 0.00001, I2 = 2%). (Fig.S3.1)

### Comparison of postoperative major complications

**Fig. 3:**
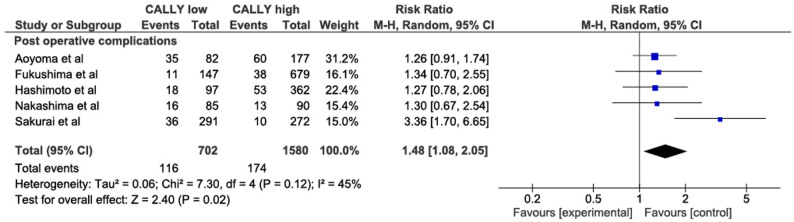
Comparison of postoperative major complications

Five studies [13, 18, 19, 21, 27] were included in the analysis, and the forest plot revealed that the low CALLY index group had a higher postoperative major complications rate than the high CALLY index group (RR = 1.48, 95% CI 1.08–2.05, *P* = 0.02, I2 = 45%; Fig. 3). Due to the moderate heterogeneity, a subgroup analysis was performed depending on the TNM stage and the CALLY cutoff value. Subgroup heterogeneity was high in the “CALLY index cutoff < 3” group (RR = 2.11, 95% CI 0.85–5.26, *P* = 0.11, I2 = 74%), which had a non-significant result. A subgroup analysis of the studies with I-III stages patients included all the studies except Sakurai et al. and revealed significant result with no heterogeneity (RR = 1.28, 95% CI 1.01–1.61, *P* = 0.04, I2 = 0%). (Fig.S3.2)

### Assessment of publication bias

**Fig. 4:**
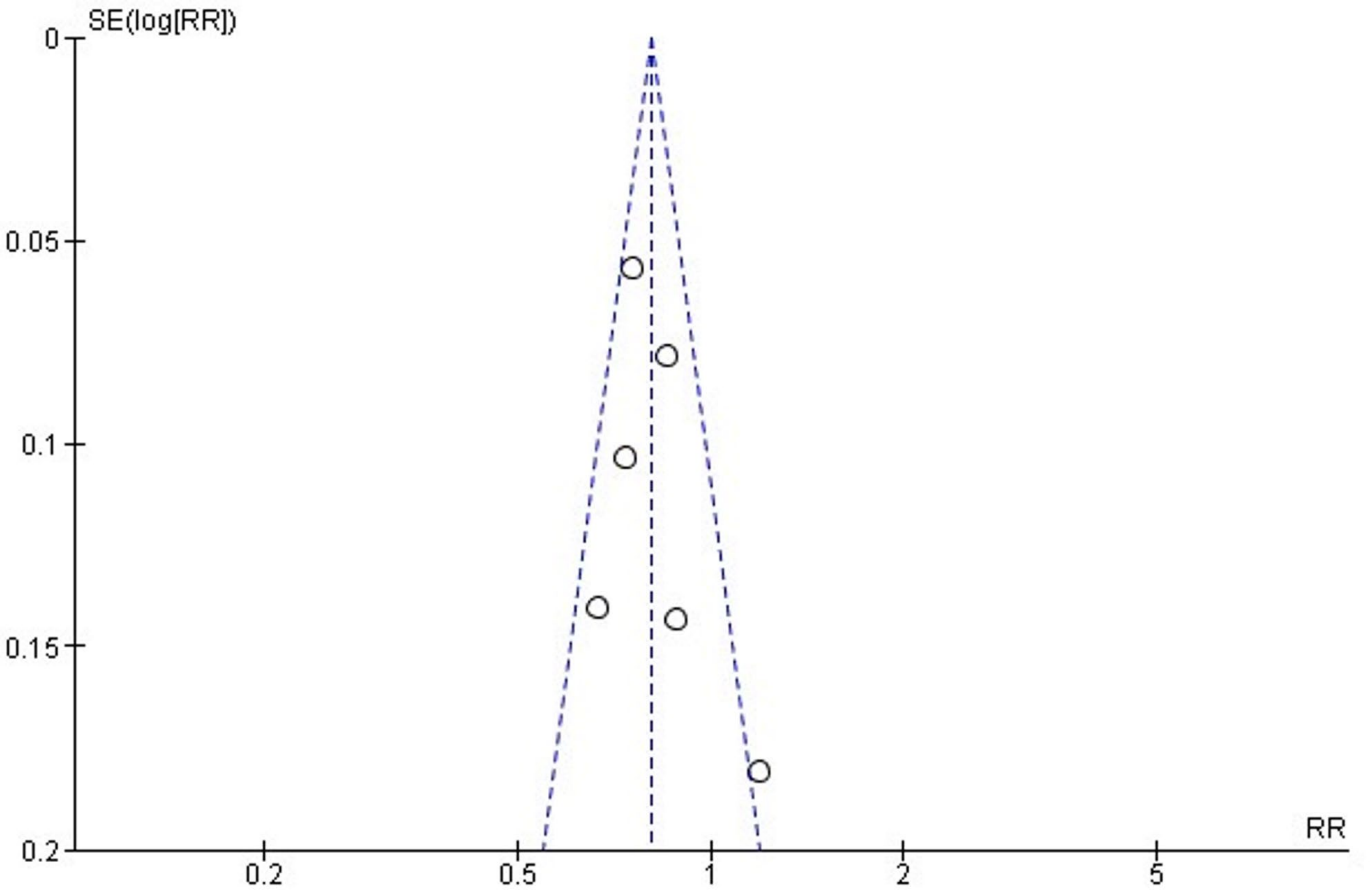
Funnel plot for publication bias assessment in gastric cancer studies

A funnel plot was used to estimate publication bias in gastric cancer studies, which showed no overt bias (Fig. 4). Egger test revealed a nonsignificant intercept indicating no significant publication bias (*p* = 0.399).

## Discussion

This systematic review and meta-analysis synthesized data from 21 studies evaluating the prognostic role of the CALLY index in GI malignancies. Given the biological heterogeneity of GI cancers, there is a growing need for prognostic markers that complement conventional staging and support individualized treatment. While the TNM classification remains the gold standard [6], its limitations are well recognized, particularly in patients with similar stages but varying outcomes due to host-related factors such as nutritional and inflammatory status [33, 34]. Consequently, composite prognostic markers integrating these parameters have gained attention.

The CALLY index combines serum albumin, total lymphocyte count, and CRP, reflecting nutritional status, immune function, and systemic inflammation. Its derivation stems from evidence that inflammation and malnutrition significantly affect cancer prognosis [35]. Inflammation, marked by elevated CRP, promotes tumor proliferation, angiogenesis, and metastasis through cytokine-mediated activation of pathways such as IL-6/STAT3 and NF-κB signaling, which enhance tumor cell survival and resistance to apoptosis [36–38]. Hypoalbuminemia indicates both systemic inflammatory burden and impaired hepatic protein synthesis, frequently associated with cancer cachexia [39]. Concurrently, reduced lymphocyte counts limit cytotoxic T-cell–mediated tumor control. Together, these alterations reflect a host microenvironment permissive to tumor progression and potentially less responsive to systemic therapies. The CALLY index therefore has multidimensional prognostic value that has been demonstrated across various malignancies, including ovarian, breast, oral, and non-small cell lung cancers, where lower values correlated with poorer survival [40–43]. However, its role in GI cancers has only recently been explored, providing the rationale for this review.

Across the 21 included studies, the CALLY index was evaluated in six different GI cancers, with OS being the most reported outcome (*n = 18*). In all studies, patients were stratified into high- and low-CALLY groups, consistently showing better survival in the high-CALLY group. However, the magnitude of effect varied, with HRs for OS ranging from 1.43 in gastric cancer to 3.86 in esophageal cancer. Among the studies reporting these HRs, the applied CALLY index cutoff values ranged from 1.12 to 2.4 [23, 32], reflecting both biological and methodological heterogeneity. This variation in cutoff thresholds, along with differences in patient populations and cancer types, may account for the observed discrepancies. These findings suggest that while inflammatory and nutritional markers hold prognostic value, their impact may differ across malignancies, necessitating further investigation.

The cutoff values used to define high versus low CALLY index varied substantially across studies (0.369–6.96), reflecting tumor-specific, population-specific, and methodological heterogeneity. Several methodological factors likely contributed to this variability. First, tumor type and included stages influence the baseline inflammation; advanced gastrointestinal and hepatobiliary malignancies, for example, are frequently associated with chronic inflammation and hepatic dysfunction [44, 45], potentially shifting thresholds. Second, population characteristics such as age distribution and prevalence of comorbidities in baseline laboratory values may influence index distribution. Third, statistical approaches differed across studies, with most deriving cutoffs using receiver operating characteristic (ROC) curve analysis, while others used previously established thresholds based on prior literature or survival rates, in addition to other statistical methods, inherently generating inconsistent thresholds.

In EC, the need for reliable prognostic biomarkers remains high due to persistently poor outcomes [2]. A prior review of inflammatory and nutrition-based biomarkers in EC patients found significant associations between multiple indices and poor prognostic outcomes, including OS, DFS, and CSS [46]. Specifically, elevated NLR, PLR, and CRP-to-albumin ratio (CAR), as well as decreased lymphocyte-to-monocyte ratio (LMR), were linked to worse prognosis. These findings highlight the role of low albumin and lymphocyte levels, along with elevated CRP levels, in poor prognosis; aligning with our observations. Notably, while none of the biomarkers in the previous study demonstrated an HR above 2 for OS, both studies in our review reported higher HRs for OS (2.31 and 3.86), suggesting the CALLY index may offer stronger prognostic value [17, 23]. This trend extended to CSS and DFS.

A similar prognostic pattern has been reported in CRC, where elevated CRP and reduced lymphocyte and albumin levels correlated with poorer survival [47, 48]. CEA remains the most recognized prognostic marker, with a meta-analysis reporting pooled HRs of 1.624 for OS and 1.453 for DFS [49]. Although we could not pool HRs for CRC due to limited studies, individual CALLY-based HRs were comparable or higher than those for CEA. These findings suggest the CALLY index may have comparable prognostic utility, though further comparison studies are needed.

As for HCC, the prognostic significance of the CALLY index has been demonstrated in patients undergoing hepatectomy or curative-intent resection for tumors exceeding Milan criteria, where lower values were associated with poorer 5-year OS and a higher risk of VER; respectively. Similarly, in TACE-treated patients, low CALLY values predicted worse OS. These findings align with growing evidence that albumin, lymphocytes, CRP, and their derived ratios play a significant prognostic role in HCC [50, 51]. Additionally, elevated CRP is tightly linked to increased interleukin-6 (IL-6), which is implicated in tumor progression through IκB kinase β/nuclear factor-κB signaling [52, 53]. Thus, systemic inflammation and nutritional status remarkably shape HCC outcomes.

This association extends to PC and CC, which are both characterized by poor survival and limited treatment options [54, 55]. In surgical patients with PC or CC, low CALLY values were linked to worse 5-year OS, CSS, and DFS. While studies specifically investigating the CALLY index in these malignancies are limited, their findings support the broader role of inflammation in cancer progression [56–59]. IL-6, for instance, acts as an autocrine growth factor in CC cell lines, cementing the link between inflammatory pathways and disease outcomes [60]. Similarly, a recent meta-analysis identified CAR as a significant prognostic marker in PC, reinforcing the relevance of combined inflammatory and nutritional indices [61]. While the CALLY index’s association with postoperative complications remains unclear, a study by Lu et al. suggests that biomarkers incorporating nutritional status, inflammation, and immune function correlate with in-hospital length of stay, pointing to potential implications for perioperative risk stratification [62].

Regarding GC, a previous meta-analysis by Li et al. found a lower CALLY index associated with poorer survival [63]. Our meta-analysis, including an additional 563 patients from Sakurai et al., reinforced this association, showing consistent improvement in 5-year OS with higher CALLY values [27]. When restricting analysis to patients undergoing gastrectomy, homogeneity was achieved, indicating greater relevance in mere surgical settings. Subgroup analyses revealed that heterogeneity stemmed primarily from advanced-stage GC (I–IV), where stage IV patients consistently had low CALLY values, likely reflecting malnutrition and hypoalbuminemia, which are linked to impaired immunity, greater chemotherapy resistance, heightened adverse effects, and reduced overall survival [64–67]. Additionally, studies using a CALLY cutoff < 3 showed inconsistent associations with OS, due to the heterogeneity of the studies in this group in terms of tumor TNM stage and treatment modality, possibly limiting prognostic reliability.

Clinically, the CALLY index may serve as an adjunct for risk stratification. Patients with low CALLY scores, could be considered at higher risk for adverse oncologic and postoperative outcomes [68]. In addition, CALLY index can predict response to different treatment modalities Inflammation-driven cytokine activation has been implicated in chemotherapy resistance and radiotherapy repone [69], while hypoalbuminemia is associated with altered drug pharmacokinetics and increased toxicity risk. Moreover, lymphopenia has been linked to impaired antitumor immune response and reduced efficacy of cytotoxic and immunomodulatory therapies [70]. These observations suggest that the CALLY index may not only predict prognosis but also reflect treatment resilience and potential response variability., possibly directing the treatment regimen [71]. In non-surgical settings, a low CALLY index may support closer follow-up, consideration of multimodal treatment approaches, or integration into composite prognostic nomograms alongside TNM staging and performance status.

Beyond prognosis, this meta-analysis is the first to assess CALLY’s association with major postoperative complications (Clavien-Dindo grade ≥ III) in GC. Patients with low CALLY values had significantly higher complication rates, aligning with evidence linking hypoalbuminemia and poor nutritional status to impaired healing and infection risk [72, 73]. Similarly, CAR has been linked to postoperative risks in GC patients [74]. In surgical candidates, preoperative identification of low-CALLY patients may justify intensified perioperative nutritional optimization, immunonutrition strategies, or enhanced postoperative surveillance, since preoperative optimization of nutritional and inflammatory status is related to reduced postoperative complications [69, 75].

Although this study demonstrated consistent prognostic significance of the CALLY index across multiple gastrointestinal malignancies, its clinical relevance should be interpreted in relation to other established inflammatory and nutritional indices. In metastatic gastric cancer, elevated neutrophil-to-lymphocyte ratio (NLR) has been associated with poorer OS (HR = 1.61, 95% CI 1.18–2.21) and a higher prognostic nutritional index (PNI) demonstrated a protective effect (HR = 0.72, 95% CI 0.53–0.97) [76], whereas pooled analysis for the CALLY index demonstrated a smaller effect size with reduced OS in the low CALLY group (RR = 0.81, 95% CI 0.74–0.87). In esophageal cancer cohorts, hazard ratios for OS reached up to approximately 3.86 [23], exceeding those reported for several individual indices, including NLR (HR = 1.43, 95% CI 1.30–1.58), platelet-to-lymphocyte ratio (PLR) (HR = 1.26, 95% CI 1.18–1.35), C-reactive protein-to-albumin ratio (CAR) (HR = 1.84, 95% CI 1.60–2.10), Glasgow Prognostic Score (GPS) (HR = 2.35, 95% CI 1.99–2.76), and reduced PNI (HR = 1.51, 95% CI 1.36–1.68) [46].

In colorectal cancer, elevated pretreatment NLR predicts worse OS (HR = 1.81, 95% CI 1.499–2.193) [77], whereas studies included in the present analysis demonstrated worse OS among patients with lower CALLY values, with hazard ratios of 0.60 (95% CI 0.39–0.94) and 0.45 (95% CI 0.36–0.56), while higher CALLY values were associated with improved OS (HR = 2.79, 95% CI 1.32–5.92). These effect sizes indicate a magnitude of prognostic association that is comparable to, and in some cohorts greater than, that reported for NLR. Likewise, CEA demonstrates modest prognostic value (pooled HR = 1.346, 95% CI 1.083–1.671) [49], whereas the CALLY index showed a stronger association with survival outcomes.

In pancreatic cancer, the CRP-to-albumin ratio (CAR) and has been associated with poorer overall survival (HR = 1.98, 95% CI 1.58–2.48) [61] and the Prognostic nutritional index (PNI) with improved OS (OR = 1.57, 95% CI 1.20–2.05) [77], whereas studies included in the present analysis demonstrated worse overall survival in patients with low CALLY values following resection (HR = 1.772, 95% CI 1.362–2.305; HR = 1.56, 95% CI 1.10–2.22), indicating a prognostic effect size comparable to CAR. Composite host-status scores such as CONUT (HR = 5.01, 95% CI 2.27–10.98) and ALBI grade (HR = 17.86, 95% CI 9.63–31.04) demonstrate larger reported effect sizes [78], likely reflecting their incorporation of hepatic functional reserve. Nevertheless, these findings collectively underscore the clinical importance of host inflammatory, nutritional, and physiologic status, factors also captured by the CALLY index, which integrates albumin, lymphocyte count, and CRP, while CONUT incorporates albumin, lymphocyte count, and cholesterol, and ALBI reflects hepatic function based on albumin and bilirubin levels.

Despite the promising findings of this study, several limitations should be considered. The predominance of retrospective cohorts introduces inherent bias in patient selection, data collection, and reporting, which may affect the robustness of the observed associations. Moreover, the lack of standardized CALLY cutoff values across included studies and the absence of a universally accepted threshold in the current clinical setting further limit generalizability and potentially impact the reliability and cross-study comparability of results. Establishing a consensus cutoff is necessary to facilitate clinical application, but from a practical standpoint, rather than advocating for a single universal cutoff, future research should prioritize external validation within tumor-specific cohorts and standardized statistical methodologies. It is worth noting that the CALLY index was not analyzed as a continuous variable in any of the included studies, which may offer more nuanced insights; while categorization enhances clinical simplicity and bedside applicability, it may result in loss of prognostic information and introduce misclassification bias, particularly near threshold values, further studies modeling the CALLY index as a continuous variable, may better explain the relationships between systemic inflammation, nutritional status, immune competence, and oncologic outcomes. Ultimately, efforts should aim to balance prognostic precision with clinical usability to facilitate broader implementation. Furthermore, the included studies did not consistently provide stratified analyses according to treatment modalities (e.g., surgical resection, locoregional therapies, or systemic therapy), precluding assessment of whether the prognostic value of the CALLY index varies across therapeutic contexts. While the CALLY index reflects host inflammatory and nutritional status, it does not incorporate tumor biology or molecular characteristics. In addition, direct comparison with other inflammatory and nutritional prognostic indices (e.g., NLR, PLR, PNI, GPS) was not feasible because standardized head-to-head analyses were not reported in the included studies.

Additionally, although studies from multiple geographic regions were included, most originated from East Asia, with the majority conducted in Japan and limited representation from other regions. This imbalance may affect the generalizability of the findings to Western and other non-Asian populations. Cancer epidemiology, tumor biology, and baseline inflammatory profiles differ across geographic regions due to variations in genetic background, environmental exposures, dietary patterns, prevalence of chronic inflammatory conditions, and healthcare systems [79]. In addition, variations in surgical techniques, perioperative management, and oncologic treatment standards across regions may further modify the prognostic performance of inflammation-based biomarkers. Therefore, while the present findings support the prognostic value of the CALLY index, external validation in ethnically and geographically diverse cohorts is essential before implementation in global practice.

In this study, although publication bias was formally assessed, the exclusion of conference abstracts and grey literature may have introduced potential reporting bias. Studies with negative or null findings are less likely to undergo full peer-reviewed publication, which could lead to overestimation of prognostic effects. Additionally, multiple included studies reported incomplete baseline data, with TNM stage or age categorized as “not otherwise specified (NOS)” or “not available (N/A).” These limitations restricted the granularity of subgroup analyses and may have contributed to residual heterogeneity. Because subgroup analyses were based on available reported data, missing covariate information could have influenced pooled estimates.

Future research should prioritize prospective, multicenter investigations to validate the prognostic utility of the CALLY index across diverse populations and establish more robust and standardized cutoff values for clinical application. Such studies should include clearly defined patient cohorts across different disease stages and treatment strategies and evaluate clinically meaningful endpoints, including overall survival, disease-free survival, and postoperative complications. Furthermore, evaluation of the CALLY index as a continuous variable, integration with tumor biological markers, and direct comparative analyses with established inflammatory and nutritional prognostic indices may further enhance prognostic accuracy and clarify its incremental clinical value.

Findings from this systematic review and meta-analysis highlight the CALLY index as a promising prognostic biomarker for GI malignancies, particularly gastric and colorectal cancers. A higher CALLY index was associated with improved OS and DFS across multiple GI cancers and a lower rate of postoperative complications in patients undergoing surgical resection. However, for certain malignancies, especially pancreatic and biliary tract cancers, the limited number of available studies limits the applicability and generalizability of these findings to those specific tumor types, and conclusions should be interpreted cautiously pending validation in larger, multicenter prospective studies. Standardization of cutoff values and validation in diverse populations are essential for optimizing its clinical application and integration into treatment decision-making.

## Supplementary Information

Supplementary Material 1.

Supplementary Material 2.

Supplementary Material 3.

## Data Availability

All data generated or analyzed during this study are included in this article, further inquiries can be directed to the corresponding author.
