# Supplementary material for: Prognostic role of C-reactive protein-albumin-lymphocyte (CALLY) index in gastrointestinal malignancies: a systematic review and meta-analysis
Source: BMC Gastroenterol. 2026 Apr 10;26:307. doi: 10.1186/s12876-026-04793-7 (PMC13181964; doi:10.1186/s12876-026-04793-7)
Supplement: Supplementary file 1 — Supplementary Material 1. [file 12876_2026_4793_MOESM1_ESM.docx]

**Detailed Search Syntax**

1. ***PubMed:***

(("C-reactive protein albumin lymphocyte index"[Title/Abstract] OR "CALLY index"[Title/Abstract] OR "CRP albumin lymphocyte index"[Title/Abstract] OR "C-reactive protein albumin lymphocyte ratio"[Title/Abstract]) AND ("gastrointestinal neoplasms"[MeSH Terms] OR "gastrointestinal cancers"[Title/Abstract] OR "gastrointestinal malignancies"[Title/Abstract] OR "esophageal neoplasms"[MeSH Terms] OR "gastric cancer"[Title/Abstract] OR "stomach neoplasms"[MeSH Terms] OR "pancreatic cancer"[Title/Abstract] OR "colorectal neoplasms"[MeSH Terms] OR “colorectal cancer”[Title/Abstract] OR "anal cancer"[Title/Abstract] OR "liver neoplasms"[MeSH Terms] OR “hepatocellular carcinoma”[Title/Abstract] OR "biliary tract cancer"[Title/Abstract] OR "cholangiocarcinoma"[Title/Abstract] OR "small intestine cancer"[Title/Abstract]))

1. ***Scopus:***

TITLE-ABS("C-reactive protein albumin lymphocyte index" OR "CALLY index" OR "CRP albumin lymphocyte index" OR "C-reactive protein albumin lymphocyte ratio") AND TITLE-ABS("gastrointestinal cancers" OR "gastrointestinal malignancies" OR "gastric cancer" OR "colorectal cancer" OR "anal cancer" OR "pancreatic cancer" OR "biliary tract cancer" OR "cholangiocarcinoma" OR "small intestine cancer" OR "hepatocellular carcinoma") OR INDEXTERMS("gastrointestinal neoplasms" OR "esophageal neoplasms" OR "stomach neoplasms" OR "colorectal neoplasms" OR "liver neoplasms")

1. ***Web of Science:***

TS=("C-reactive protein albumin lymphocyte index" OR "CALLY index" OR "CRP albumin lymphocyte index" OR "C-reactive protein albumin lymphocyte ratio") AND TS=("gastrointestinal neoplasms" OR "gastrointestinal cancers" OR "gastrointestinal malignancies" OR "esophageal neoplasms" OR "gastric cancer" OR "stomach neoplasms" OR "pancreatic cancer" OR "colorectal neoplasms" OR "colorectal cancer" OR "anal cancer" OR "liver neoplasms" OR "hepatocellular carcinoma" OR "biliary tract cancer" OR "cholangiocarcinoma" OR "small intestine cancer")
