# Supplementary material for: Prognostic role of C-reactive protein-albumin-lymphocyte (CALLY) index in gastrointestinal malignancies: a systematic review and meta-analysis
Source: BMC Gastroenterol. 2026 Apr 10;26:307. doi: 10.1186/s12876-026-04793-7 (PMC13181964; doi:10.1186/s12876-026-04793-7)
Supplement: Supplementary file 2 — Supplementary Material 2. [file 12876_2026_4793_MOESM2_ESM.docx]

**Modified Newcastle-Ottawa Quality Assessment Scale**

A study can be awarded with a maximum of 1 star for each numbered item within the Selection and Comparability categories. A maximum of two stars can be given for comparability.

| No. | Criterion |  | Score  ☆ = 1 |
| --- | --- | --- | --- |
| **Selection (max 1 star per criterion)** | | | |
| 1 | Representativeness of the exposed cohort | 1. all eligible participants during the study period were included in the study ☆ 2. a random selection of eligible participants during the study period were included in the study ☆ 3. a non-random selection of eligible participants during the study period were included in the study 4. no description of how the cohort was selected |  |
| 2 | Selection of the Non-Exposed Cohort | 1. drawn from the same source population ☆ 2. drawn from a different source population 3. no description of how the non-exposed was selected |  |
| 3 | Ascertainment of Exposure | 1. secure record ☆ 2. reported by physician ☆ 3. written self-report 4. no description |  |
| 4 | Demonstration That Outcome of Interest Was Not Present at Start of Study | 1. yes ☆ 2. no, or unclear |  |
| **Comparability (max 2 stars can be given)** | | | |
| 1 | Comparability of Cohorts on the Basis of the?? | 1. study reports / controls for age ☆ 2. Age not reported |  |
| 2 | Comparability of Cohorts on the Basis of the?? | 1. study reports / controls for TNM staging ☆ 2. TNM staging not reported |  |
| **Outcome (max 1 star per criterion)** | | | |
| 1 | Assessment of Outcome | 1. independent blind assessment stated ☆ 2. record linkage ☆ 3. self-report 4. no description |  |
| 2 | Was Follow-Up Long Enough for Outcomes to Occur | 1. yes (≥ 6 months follow up) ☆ 2. no (< 6 months) |  |
| 3 | Adequacy of Follow Up of Cohorts | 1. complete follow up – all subjects accounted for ☆ 2. subjects lost to follow up unlikely to introduce bias (>95% follow up, or description provided of those lost) ☆ 3. follow up rate ≤95%, and no description of those lost provided 4. no statement |  |
|  | | SCORE: | |

**Detailed NOS Scores**

| **Author** | **Selection** | **Comparability** | **Outcome** | **Total Score** |
| --- | --- | --- | --- | --- |
| Aoyama + Hashimoto et al. | ★★★★ | ★★ | ★★★ | 9 |
| Aoyoma + Maezawa et al. | ★★★★ | ★★ | ★★★ | 9 |
| Feng et al. | ★★★★ | ★★ | ★★★ | 9 |
| Fukushima et al. | ★★★★ | ★★ | ★★★ | 9 |
| Furukawa et al. | ★★★★ | - | ★★★ | 7 |
| Hashimoto et al. | ★★★★ | ★★ | ★★★ | 9 |
| Iida et al. | ★★★★ | ★★ | ★★★ | 9 |
| Kawahara et al. | ★★★★ | ★★ | ★★★ | 9 |
| Kosaka et al. | ★★★★ | ★ | ★★★ | 8 |
| Ma et al. | ★★★★ | ★★ | ★★★ | 9 |
| Matsui et al. | ★★★★ | - | ★★★ | 7 |
| Müller et al. | ★★★★ | ★ | ★★★ | 8 |
| Nakashima et al. | ★★★★ | ★★ | ★★★ | 9 |
| Okugawa et al. | ★★★★ | ★★ | ★★★ | 9 |
| Sakurai et al. | ★★★★ | ★ | ★★★ | 8 |
| Shiraishi et al. | ★★★★ | - | ★★★ | 7 |
| Takeda et al. | ★★★★ | ★★ | ★★★ | 9 |
| Tsunematsu et al. | ★★★★ | ★★ | ★★★ | 9 |
| Yang et al. | ★★★★ | ★★ | ★★★ | 9 |
| Yasuda et al. | ★★★★ | - | ★★★ | 7 |
| Zhang et al. | ★★★★ | ★★ | ★★★ | 9 |
