# Supplementary material for: Prognostic role of C-reactive protein-albumin-lymphocyte (CALLY) index in gastrointestinal malignancies: a systematic review and meta-analysis
Source: BMC Gastroenterol. 2026 Apr 10;26:307. doi: 10.1186/s12876-026-04793-7 (PMC13181964; doi:10.1186/s12876-026-04793-7)
Supplement: Supplementary file 3 — Supplementary Material 3. [file 12876_2026_4793_MOESM3_ESM.docx]

**Fig.S3.1:** Subgroup Analysis of the Comparison of the Patients’ 5-year Overall Survival (OS)

**
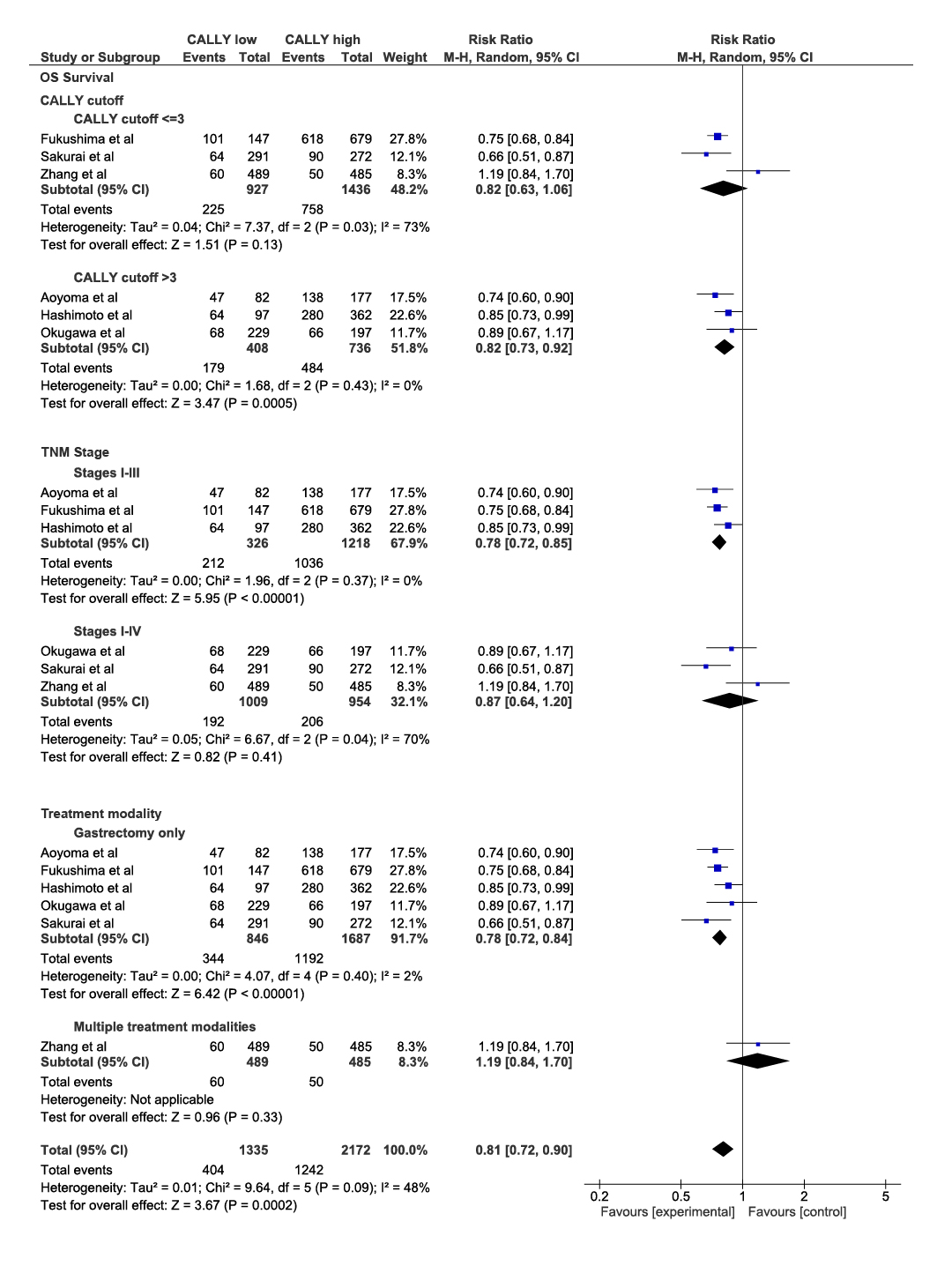
**

**Fig.S3.2:** Subgroup Analysis of the Comparison of Postoperative Major Complications

**
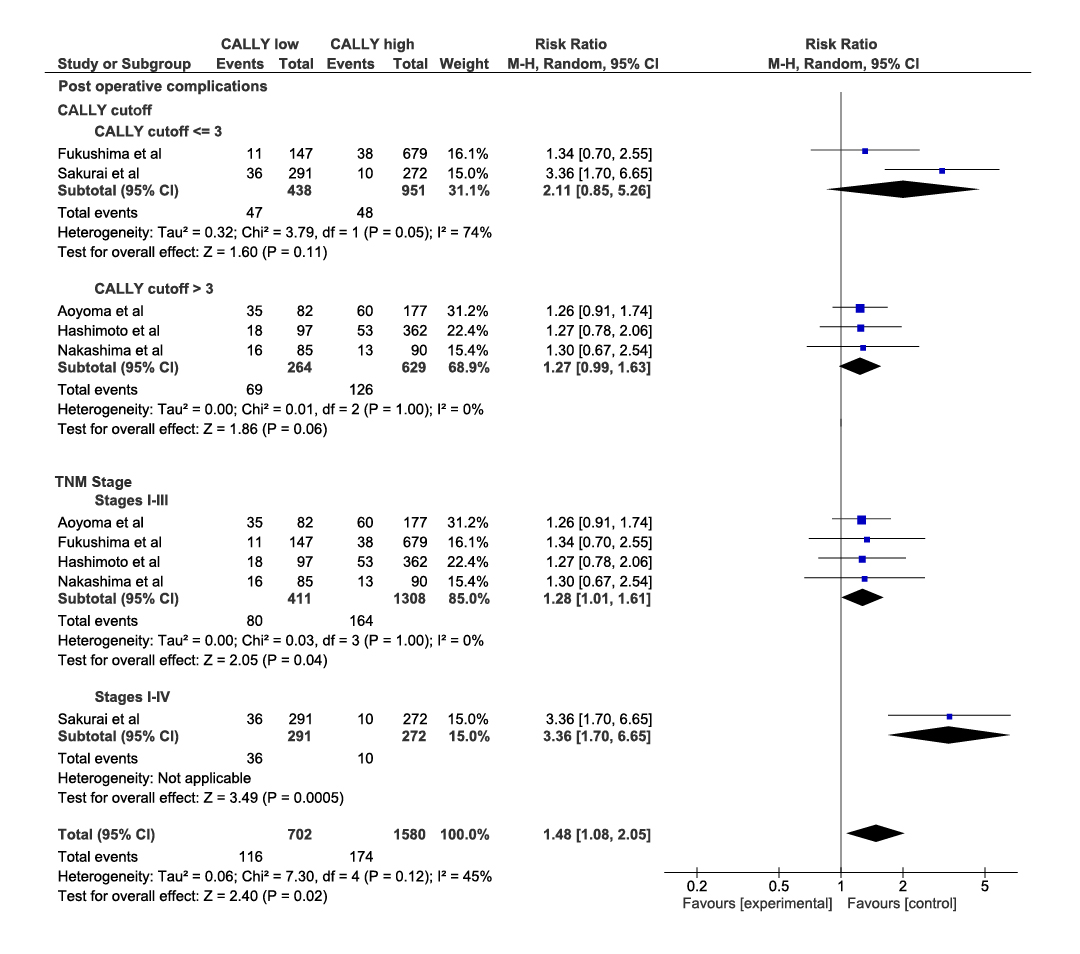
**
